# Superconducting Materials and Devices Grown by Focused Ion and Electron Beam Induced Deposition

**DOI:** 10.3390/nano12081367

**Published:** 2022-04-15

**Authors:** Pablo Orús, Fabian Sigloch, Soraya Sangiao, José María De Teresa

**Affiliations:** 1 Instituto de Nanociencia y Materiales de Aragón (INMA), CSIC-Universidad de Zaragoza, 50009 Zaragoza, Spain; porus@unizar.es (P.O.); fsigloch@unizar.es (F.S.); sangiao@unizar.es (S.S.); 2 Departamento de Física de la Materia Condensada, Facultad de Ciencias, Universidad de Zaragoza, 50009 Zaragoza, Spain; 3 Laboratorio de Microscopías Avanzadas (LMA), University of Zaragoza, 50018 Zaragoza, Spain

**Keywords:** superconductivity, nanofabrication, focused ion beam, focused electron beam, tungsten, niobium, superconducting vortex, SQUID, superconducting proximity effect

## Abstract

Since its discovery in 1911, superconductivity has represented an equally inciting and fascinating field of study in several areas of physics and materials science, ranging from its most fundamental theoretical understanding, to its practical application in different areas of engineering. The fabrication of superconducting materials can be downsized to the nanoscale by means of *Focused Ion/Electron Beam Induced Deposition*: nanopatterning techniques that make use of a focused beam of ions or electrons to decompose a gaseous precursor in a single step. Overcoming the need to use a resist, these approaches allow for targeted, highly-flexible nanopatterning of nanostructures with lateral resolution in the range of 10 nm to 30 nm. In this review, the fundamentals of these nanofabrication techniques are presented, followed by a literature revision on the published work that makes use of them to grow superconducting materials, the most remarkable of which are based on tungsten, niobium, molybdenum, carbon, and lead. Several examples of the application of these materials to functional devices are presented, related to the superconducting proximity effect, vortex dynamics, electric-field effect, and to the nanofabrication of Josephson junctions and nanoSQUIDs. Owing to the patterning flexibility they offer, both of these techniques represent a powerful and convenient approach towards both fundamental and applied research in superconductivity.

## 1. Introduction

### 1.1. Superconductivity

Superconductivity is an electronic state of matter attained below certain critical values of temperature, magnetic field, and applied current. In this state, the resistance to the passage of an electric current exhibited by the material is zero. In the case of isotropic *s*-wave superconductivity, the microscopic justification of this behavior is provided by the Barden-Cooper-Schiefer theory [1], accounted for by the transport of charge via bosonic Cooper pairs; while the macroscopic framework, built upon the definition of a superconducting wavefunction that characterizes the superconducting state, comes from the Ginzburg–Landau theory [2].

In a three-dimensional phase diagram, the superconducting state can be thought of as bound by fundamentally three experimentally controllable parameters: the temperature, the magnetic field, and the driving current. Different materials exhibit characteristic, critical values for each of these parameters: the critical temperature, $T_{c}$, typically used as the trademark “signature” of each superconductor, the critical magnetic field, $B_{c}$, and the critical current density, $J_{c}$. The actual values of these critical variables depend on the value the others take when the measurement is performed, achieving their nominal value only when the rest of variables are set (or extrapolated) to zero. Other spatial parameters of interest are the penetration depth λ, which quantifies the extent to which an externally applied magnetic field can enter the material, and the coherence length ξ, which reflects the allowed spatial variations of the electron density.

Depending on how they respond to an externally-applied magnetic field, superconducting materials may be classified into type-I and type-II superconductors. Type-I materials exhibit what one might think of as the “standard” behavior against such a field: below the critical temperature, if the material is subject to an external magnetic field, the non-dissipative nature of the superconducting state allows for the appearance of virtually unbound screening supercurrents that oppose the incoming magnetic flux. As such, the material maintains perfect diamagnetism as long as the magnetic field is kept below its critical value (which would drive the material back into the normal state): in a type-I superconductor, all magnetic field flux lines are completely expelled from the bulk of the material while it is kept in the superconducting state. This phenomenon is known as Meissner effect [3].

On the other hand, type-II superconductors are characterized by the existence of a mixed state between the normal and Meissner phases, bound by the so-called lower and upper critical fields ($B_{c1}$ and $B_{c2}$, respectively), in which the magnetic field flux lines can penetrate the material. However, the penetration of the flux takes place in a quantized form: the incoming magnetic flux is redistributed in an array of individual flux quanta $\Phi_{0}$ ($\Phi_{0}=h/2e=2.07\times 10^{-15}$ Wb) that pierce the material. Each flux quantum threads the superconductor through a locally normal region, oriented along the direction of the field, and screened from the rest of the bulk by a circular supercurrent. These entities are named *superconducting vortices* and are typically called Abrikosov vortices (or Pearl vortices in very thin films) [4,5,6].

The dissipationless regime provided by superconductors exhibits tremendous potential for many technological applications. Since the restrictive condition of attaining low critical temperatures severely hampers the applicability of the materials for macroscopic current transport, the search for room-temperature superconductors represents one of the most compelling ongoing quests in physics and materials science [7,8]. Still, the superconducting regime can be practically exploited for the generation of stable magnetic fields, finding application to provide such required fields in syncrotron facilities [9] and particle colliders [10], in magnetic resonance imaging equipment [11], and in plasma confinement systems [12]. Nanopatterned superconductors are no exception to this trend, representing an inciting field of study and application.

### 1.2. Nanopatterning Superconducting Materials Using Focused Beams

Scaling superconducting materials down to micro- and nanometric sizes provides with an additional element of interest—by reducing the characteristic dimensions of the material to values comparable to those of its characteristic lengths, new effects that were initially absent in the macroscale, do emerge [13]. These include modifications in the values of the critical parameters and in the transport properties of the material or device. The appearance, investigation, and exploitation of such effects are foundational to the development of quantum technologies, on which superconducting nanodevices represent a particularly blooming research field. Remarkable examples of quantum technology in the form of superconducting nanodevices include magnetic sensors in the form of superconducting quantum interference devices (SQUIDs) [14], single-photon detectors [15], quantum bits [16], and quantum switches [17].

The constant development of ever-improving nanofabrication techniques is a perennial need for both the industry and the research communities. Three widely-known and amply utilized nanopatterning techniques are optical lithography, electron beam lithography, and focused ion/electron beam induced deposition. Optical lithography excels with its high throughput [18], while electron beam lithography is capable of achieving remarkable resolution in tailored designs [19]. Both of them, however, exhibit several significant drawbacks: the unavoidable need for a resist, which needs to be exposed to the corresponding radiation and then revealed by means of a chemical agent [20,21]; the difficulties that arise in the patterning of three-dimensional structures; and its complicated use on unconventional substrates such as cantilevers.

*Focused Ion Beam Induced Deposition* (FIBID) and *Focused Electron Beam Induced Deposition* (FEBID) are two very similar nanopatterning techniques that make use of a focused beam of charged particles, either ions or electrons, to induce the decomposition of a precursor material in gas form. The growth is performed in a single step, without the need to use a sacrificial resist; and the beam can be freely steered to trace any shape or pattern introduced by the user. This flexibility comes at the cost of a reduced throughput when compared to the two previously mentioned nanolithography techniques, particularly optical lithography. Depending on the species utilized and on the growth conditions (i.e., the operating parameters of the corresponding beam, mostly), typical achievable lateral resolutions are in the ranges of 20 nm to 30 nm for Ga${}^{+}$ ions, and around 10 nm for electrons and He${}^{+}$ ions [22,23].

The technique is also highly flexible in terms of the requirements imposed to the substrate where the deposition is to be carried out. While “conventional” Si and SiO${}_{2}$ samples are commonplace, since the beam can be freely steered to the point of interest, FIBID and FEBID also permit material growth in more geometrically complex, non-flat substrates, that would prove cumbersome or unsuitable for other deposition techniques. An example of such an application is the electron-beam induced deposition of magnetic nanowires at the apex of the tips of atomic force microscopy cantilevers, which effectively functionalizes them for the purposes of magnetic force microscopy [24,25].

Following on with the previous example, in focused beam induced deposition, the beam can be steered in such a way that the growth is performed in a continuous way over the same spot, inducing further deposition over previously grown material. Such an approach allows for effective patterning of 3D nanostructures, generally in the form of nanopillars [26]. 3D nanopatterning opens the door to investigate the differences that arise in the composition, microstructure, and electrical response of the material, owing to the contrast existing in the two deposition processes [27]; as well as to deposit functional nanostructures with complex 3D geometry [28,29,30].

The versatility and ease of applicability of both FIBID and FEBID have solidly cemented both techniques as powerful tools within the nanofabrication community. They have seen use in various applications, including targeted growth of functional materials [31], optical-lithography mask repair [32,33], and circuit editing [34].

In the following section, a summary of the working principles that govern the growth mechanism by FIBID and FEBID is presented, followed by a review on relevant published works that report the deposition of superconducting materials using these techniques. For further insight on other non-superconducting materials nanopatterned by FEBID and FIBID not discussed here, readers are invited to refer to our previous work on the matter in [22,23]. Next, the potential of the technique is showcased by reviewing specific examples that exploit its capability to investigate nanoscale physical phenomena in the field of superconductivity and to fabricate functional nanodevices.

## 2. Growth of Superconducting Materials by FIBID/FEBID

### 2.1. Principles of the Deposition Process

**Figure 1:**
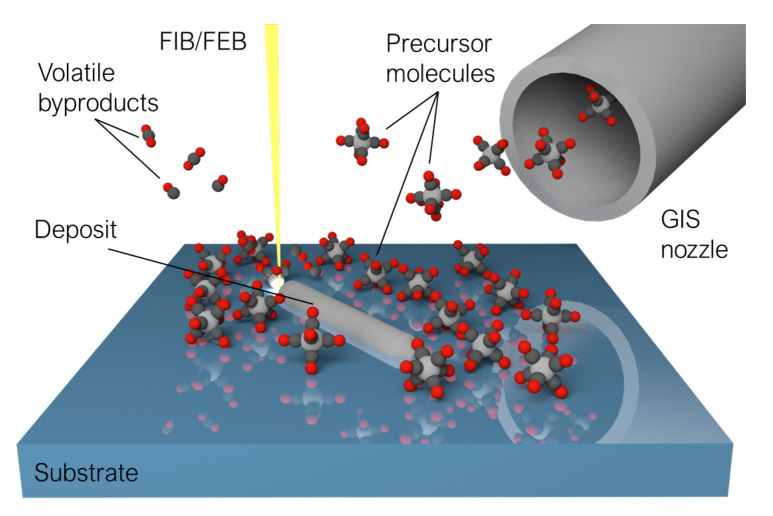
The FIBID/FEBID procedure. The beam is scanned over the surface of the substrate, decomposing the adsorbed precursor molecules on its path. Reprinted from [23], with permissions from IOP Publishing. *©* IOP Publishing Ltd. 2020.

Conceptually, both FIBID and FEBID share the same operating principle: *a precursor material in gas phase, adsorbed on a substrate, can be decomposed into volatile and non-volatile constituents by scanning a focused beam of charged particles over it*. The beam can be precisely focused and steered to scan the surface of the substrate following any pattern defined by the user, and the irradiation results in the non-volatile constituents forming a deposit whose shape matches that of said pattern (Figure 1) [31,35,36].

To perform FIBID/FEBID, the instrument that is to be utilized must be fitted with at least three fundamental constituents: a vacuum chamber where the deposition procedure is to be carried out, a focused beam of charged particles (including the source from where they are to be extracted, and the arrangements of lenses and apertures required to steer and focus the beam), and a Gas Injection System (GIS), fitted with reservoir(s) of the precursor material(s), and (at least) a nozzle used to deliver them.

One critical parameter that must be gauged when implementing a focused beam for the purposes of nanofabrication is the *spot size*, which quantifies the absolute minimum size the beam can be focused to, and therefore sets the ground for the resolution limit of the technique (which, in general, is slightly worse). Electron beams typically make use of field-emission sources, while the most commonly used ionic species for the purposes of FIBID are Ga${}^{+}$ ions, extracted from liquid metal ion sources, and He${}^{+}$ and Ne${}^{+}$ ions, obtained from gaseous ion sources. Ga${}^{+}$, He${}^{+}$, and Ne${}^{+}$ are typically chosen over other chemical elements because these sources can be engineered for these specific species to provide sufficiently reduced spot sizes, required for the purposes of nanopatterning [23]. The specific value of the spot size for each of these beams depends on the operating conditions, but representative values are in the range of 5 nm for Ga${}^{+}$ ions [35], 1 nm for electrons [37], and 0.25 nm for He${}^{+}$ ions [38].

A commonly found, commercially available architecture is that of an FIB/SEM microscope: an instrument housing both a Ga${}^{+}$ FIB and an FEB placed at an angle with respect to each other [39]. The main advantage of this configuration is that the sample can be simultaneously imaged with the FEB and processed with the FIB when it is positioned at the convergence point of two beams. The usage of two different beams is mainly motivated by the fact that imaging with Ga${}^{+}$ ions is often counter-productive due to the relatively high milling rate and substrate damage induced by this ionic species [35]. On the other hand, He${}^{+}$ imaging does not significantly damage the sample, and as such He${}^{+}$ FIBs are typically found in dedicated equipment, where the same beam is utilized for both high-resolution imaging and patterning [40].

The growth of a nanostructure by either FIBID or FEBID can be summarized as follows:

1. The substrate where the feature is to be deposited is positioned in an adequate orientation relative to the direction of incidence of the beam. In most cases, such an orientation is that of normal beam incidence, although some specific processes might call for angled irradiation.
2. Making use of the GIS nozzle valve, which must be located in close proximity to the substrate (typically in the order of 100 μm), a gaseous precursor material is released into the chamber. The precursor molecules adsorb on the substrate, constituting a molecular monolayer in dynamical equilibrium—as adsorbed molecules exceed their residence time and are pumped away by the vacuum system, they are promptly replaced by fresh molecules coming from the GIS.
3. The focused beam is then scanned over the substrate, following a pattern defined by the user and decomposing the adsorbed molecules in its path. The GIS nozzle is kept open until the irradiation ends in order to replenish the precursor monolayer as it is being depleted [41].
4. When the irradiation is completed, the GIS nozzle is closed, and the vacuum system evacuates any gaseous remnants in the chamber.

The deposition of the material is achieved in both processes through a localized energy transfer from the incoming charged particles to the precursor molecules, typically mediated by the substrate. As the accelerated ions or electrons impinge on the substrate, several particles are emitted from the substrate, whose number and nature vary depending on the characteristics of the beam used to perform the irradiation.

This energy transfer may occur via elastic collisions of the charged particle with the atoms in the substrate, or by inelastic scattering events that result in secondary excitations being induced in the substrate. In the context of FEBID/FIBID, the main difference between ions and electrons for the purposes of growth and processing resides in their mass, which makes up for different beam-substrate interactions [42,43]. Owing to their comparatively large mass, impinging ions lose energy via collisions more significantly than electrons, inducing collision cascade events among the atoms in the substrate that eventually result in substrate amorphization [44], sputtering (achieved when the energy transferred to a surface atom exceeds its binding energy), and ion implantation [35]. All three of these are side effects characteristic to Ga${}^{+}$ FIBID, greatly reduced in He${}^{+}$ FIBID due to the lighter mass of those ions [40], and absent in FEBID, where inelastic events dominate [42]. FIB irradiation also results in the emission of backscattered ions [43], which are also evidently absent in FEB procedures.

Common to both procedures, and at the heart of their working principle, are *secondary electrons*, emitted in close proximity to the irradiation point as secondary excitations induced by electronic energy losses [35]. Named in such a way to differentiate them from the incoming or *primary* beam, these secondary electrons have typical energies in the order of eV, while the particles in the primary radiation are accelerated to the order of keV. The dissociation of the adsorbed precursor molecules is achieved with most efficiency at precisely these low energies characteristic to the secondary electrons [45]. Thus, the energetic primary beam does not directly interact with the adsorbed precursor molecules in a significant way whatsoever, but rather acts as a source for secondary electrons, which dominate the deposition process by transferring their energy to the precursor molecules and separating them into the aforementioned volatile and non-volatile constituents [31]. Other particles emitted by inelastic scattering include backscattered electrons and second-order secondary electrons, both of which can be exploited for imaging purposes in SEM microscopy.

One remarkable aspect of FIBID procedures is the delicate interplay that takes place between deposition and sputtering mechanisms. While the operating conditions may be adjusted to ensure that one of the mechanisms predominates over the other, both of them do occur simultaneously during irradiation. A signature of this occurrence is that nanostructures grown by FIBID typically appear slightly “embedded” on the surface of the substrate [46], as a consequence of the material removal that occurs while the precursor is being decomposed (and contrary to those grown by FEBID, whose cross-sectional views show that the substrate surface remains flat, with the deposit simply growing on top of it).

The consequences of this interplay are of particular interest in the case of He${}^{+}$ FIBID, where, owing to both the small probe size and to the sharp, narrow shape interaction volume of He${}^{+}$ ions in an FIB, each reaction induced with a He${}^{+}$ FIB takes place in a highly localized way, significantly higher than its Ga${}^{+}$ counterpart [47]. A particularly remarkable example of this simultaneous milling/deposition effect is observed in the vertical growth of 3D hollow nanopillars by He${}^{+}$ FIBID [48], which will be presented in Section 2.2.1.

In both FIBID and FEBID, but particularly relevant for the latter, where the electrons lack the necessary mass to induce milling in the material, another crucial balance that must be gauged and optimized is that of the beam with the precursor gas. Depending on the relative rates of precursor replenishment and precursor decomposition, two regimes can be distinguished: the *electron-limited regime*, where the gaseous precursor gets replenished at a rate that exceeds that at which the beam can decompose it; and the *precursor-limited regime*, where, as the name implies, the decomposition speed dominates and the growth is reigned by the amount of precursor available for decomposition [22]. This balance does influence the nature of the deposit and must be taken into account when optimizing the process.

Valuable insight on the expected behavior of the beam-substrate interactions, including their projected trajectories, energy transfer, and subsequent expected deposition yield, can be gained by means of numerical simulations based on theoretical models. In both FIBID and FEBID models, secondary electron trajectories are predicted using the Monte Carlo computational method [49,50], with most models assuming a dependence of the particle trajectory on elastic interactions only, and that incoming particles lose energy in a continuous way as they describe a trajectory within the substrate [42].

Concerning FIBID, *Transport of Ions in Matter* (TRIM) is a software that employs the Monte Carlo approach to describe the trajectories of ions in a substrate as a series of elastic interactions that together define a collision cascade; whose specifics depend on both the nature of the beam and the substrate [51]. Available as an open source software piece named SRIM (*Stopping and Range of Ions in Matter*), TRIM permits a significantly reliable estimation of the expected interaction volume of an FIB with the substrate [52]. In parallel, the software *CASINO*, which is also freely available, exploits the Monte Carlo to predict electron trajectories in a substrate [53]. Such a tool finds application not only in FEBID processes, but also in electron based lithography and spectroscopy procedures as well.

Other numerical methods employed in FIBID studies, all of which build on TRIM or SRIM, include *IONiSE* [54], which predicts secondary electron emission induced by a He${}^{+}$ FIB, *EnvizION* [55], centered in mass transport and growth rate in He${}^{+}$ FIBID processes, *TRIDYN* [56] which takes dynamical compositional changes into account, as the substrate is chemically modified by the impinging ions, and *TRI3DYN*, an expansion of TRIDYN that allows for path simulations in three dimensions [57].

### 2.2. Precursor Materials

The feasibility of an FIBID/FEBID procedure is entirely dependent on the availability of a precursor material that contains the element of interest while being suitable for decomposition under an electron or ion beam [58]. As such, it is therefore not possible to pattern any given compound known to display superconductivity using these techniques—instead, the search for superconductivity using FIBID/FEBID is rather tackled by varying the growth conditions and using different (and newer) precursors, investigating whether the deposited material exhibits superconducting behavior thereafter.

In the following sections, a review on the published work on the deposition of superconducting devices of various materials is presented.

#### 2.2.1. Tungsten

Tungsten-carbide (W-C) nanodevices fabricated by the focused beam-induced deposition of the tungsten hexacarbonyl precursor material (W(CO)${}_{6}$), can be considered the flagship of these growth techniques for superconducting materials, particularly in the case of ion-induced deposition.

Bcc-crystalline tungsten becomes superconducting at the inconveniently low temperature of 15 mK [59]. Notwithstanding, the tungsten-based material fabricated by FIBID or FEBID, which exhibits a significant amount of carbon in terms of atomic composition, does show an enhanced critical temperature in the 4 K to 5 K range. Combined with the flexibility and patterning capabilities of focused beam-induced growth, the deposition of W(CO)${}_{6}$ does represent a powerful tool for the fabrication of targeted superconducting nanodevices.

**Figure 2:**
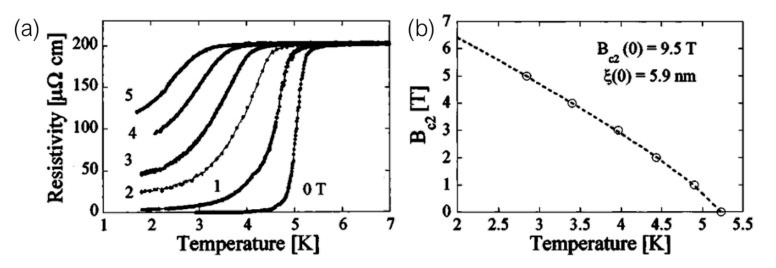
First reported experimental evidence of superconductivity in W-C nanowires prepared by Ga${}^{+}$ FIBID. (**a**) Superconducting transition at $T_{c}=5.2$ K. (**b**) Dependence of the upper critical field with temperature. Reprinted from [60], with permissions from AIP Publishing.

The first published report of the occurrence of superconductivity in a material prepared by FIBID dates back to 2004, when Sadki et al. [60] showed superconducting behavior in a W-C compound obtained by Ga${}^{+}$ FIBID of W(CO)${}_{6}$ (Figure 2). Exhibiting a metallic atomic content of around 40%, and with significant amounts of implanted Ga (20%), the non-dissipative state was detected in a nanowire below a critical temperature of 5.2 K ± 0.5 K.

**Figure 3:**
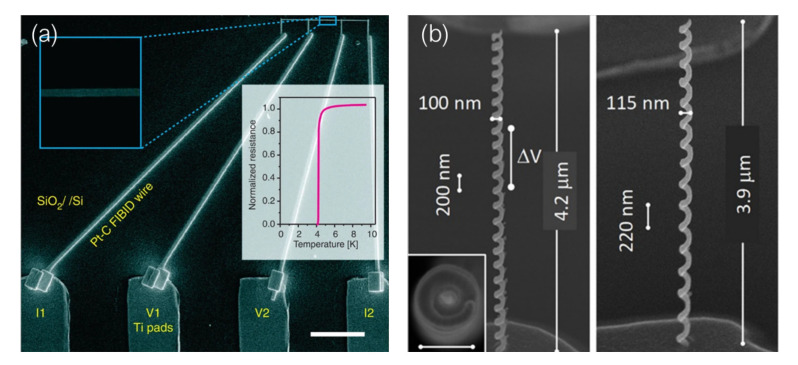
(**a**) Superconducting in-plane W-C nanowire (50 nm in width), nanopatterned by Ga${}^{+}$ FIBID. Scale bar: 5 μm. Reprinted from [65], with permissions from Springer Nature. (**b**) Superconducting 3D W-C nanohelices nanopatterned by He${}^{+}$ FIBID. Scale bar in inset: 100 nm. Reprinted from [30] with permissions from ACS Publications (https://pubs.acs.org/doi/abs/10.1021/acs.nanolett.9b03153, accessed on 15 March 2022). Further permissions related to the material excerpted should be directed to the ACS.

Since then, the Ga${}^{+}$ FIBID community has expanded on the investigation of the tunability and applicability of this superconducting material, with a wide variety of reports available in the literature [61,62,63,64,65,66,67,68,69]. While there are significant variations in the reported values of the metallic atomic content (between 20% and 50%), depending on both the deposition parameters and the technique utilized to determine it, the critical temperature is consistently found in the vicinity of 4.5 K (Figure 3a). The specific component ratio varies slightly between reports, but as representative examples, some reported values of the W:C:Ga:O ratio in terms of atomic percentage are 53:34:11:2 [63], 48:30:16:6 [64], and 40:43:10:7 [65]. Some authors have found some strategies to tune $T_{c}$ by post-growth Ga irradiation [70], and co-deposition with a C-based precursor [71]. Remarkably, the latter also report lack of superconducting behavior when the atomic W content is reduced below 17.5%, down to an insulating response when that amount is further reduced below 16%.

Most authors report the material to be amorphous, or exhibiting a microstructure of very small nanocrystallites (with sizes of around 1 nm or below), a relatively frequent microstructure also found in Pt FEBID nanostructures [72]. On this matter, care should be taken in interpreting these results, as the distinction between amorphous and nanocrystalline microstructure may become somewhat blurred, as it is harder to assess the potential crystallinity of clusters so reduced in size.

The microscopic origin of superconductivity in Ga${}^{+}$ FIBID W-C is, at the time of writing, a matter of debate, with diverging hypotheses among different authors. Li et al. [59] reported in 2008 a systematic study on the material properties with the ion beam current utilized to induce the deposition of 45 μm-long nanowires. The tungsten, carbon, and gallium contents are found to significantly depend on it, with both W and Ga concentrations increasing with increasing current, and the C concentration decreasing with higher ion beam currents. The value of $T_{c}$, however, presents a non-monotonic dependence with the current, first increasing with increasing current, up to a significant figure of 6.2 K at 50 pA, and then decreasing again when the ion beam current is further increased. Whereas W-C deposits were first described within *s*-wave type-II superconductivity [73] some authors have recently reported anisotropic superconductivity features [74].

As a general consideration for both these specific reports and those that follow in this review, it must be kept in mind that beam current is far from being the sole parameter in determining the nature of the deposits—rather, the operating conditions of such procedures are better thought of as a set of intertwined parameters, the most relevant of which are the acceleration voltage, the beam current, and dose, and the dwell time. The primary task of FIBID/FEBID experimentalists is thus to find and optimize a set of these operating parameters, and then assess how the deposition depends on them by systematic studies as the one described above.

When inducing the deposition using a He${}^{+}$ FIB, the material is also superconducting, respectively exhibiting a critical temperature range and W atomic content of 6.2 K to 7.1 K and up to 70% in 3D nanostructures [27,30] (Figure 3b), and 3 to 4 K and 20% in in-plane nanostructures [75]. The reported W:C:O component ratios in terms of atomic percentage are 72:20:8 for 3D growth [27], and 20:40:20 to 15:70:15 for in-plane growth. Even though the deposition of both materials is carried out in a conceptually equivalent manner, the differences that exist between Ga${}^{+}$ and He${}^{+}$ ions make up for significantly different growth conditions, and subsequently, different compositional and electrical properties of the deposited material. In-plane nanowires can be patterned down to a width of 10 nm (close to the coherence length of the material), virtually unattainable in a single step Ga${}^{+}$ FIBID procedure [75]. The superconducting state, however, is observed only in nanowires with widths of 20 nm and above, in the vicinity of which the transition is comparatively broad, a behavior ascribed to quantum phase slips akin to those observed in narrow nanowires of other materials.

Based on the $T_{c}$ differences with pure, crystalline W, early reports on the W-C material obtained by Ga${}^{+}$ FIBID tended to associate the occurrence of superconductivity with the lack of crystalline structure, while a justification based on the presence of implanted Ga was also proposed. However, the development and application of He${}^{+}$ FIBID have shown that the W-C material (which, when grown using that technique, is crystalline and lacks any Ga content whatsoever) is still superconducting. Therefore, none of these proposed reasons can fully account for the phenomenon, or be the *sole* reason for its appearance. At the time of writing, a plausible hypothesis that is compatible with the present data is that of the co-existence of two different superconducting phases in the material: one that is micro-structurally amorphous (typically obtained using Ga${}^{+}$ FIBID), and one that exhibits a crystalline structure (obtainable using He${}^{+}$ FIBID). In any case, further efforts, both in the experimental and theoretical fronts, need to be taken to fully account for the phenomenon.

One consequence of the growth and milling mechanisms induced by the FIB occurring simultaneously may be directly observed in the growth of vertical nanopillars, where a highly focused He${}^{+}$ beam that is constantly irradiating a fixed position, locally removes material while promoting vertical growth of a nanostructure, yielding a tube-like, hollow pillar nanostructure [48]. This effect was practically demonstrated in superconducting W-C hollow nanopillars grown via He${}^{+}$ FIBID first by Kohama et al. [76] and then further investigated by Córdoba et al. [27].

Additionally related to 3D He${}^{+}$ FIBID deposition, Córdoba et al. reported the growth of 3D nanohelices, achieved by scanning a series of circles arranged in the same location: by combining optimized growth parameters with an arrangement of irradiation spots in such a way that adjacent irradiation spots exhibit sufficient overlap, continuous vertical growth occurs as the circles are sequentially scanned [30]. In comparison with the previously discussed nanopillars, the onset of the dissipative state is enhanced in up to 35%, suggesting that the helical geometry forces a stronger pinning effect in the superconducting vortices that appear in presence of an external magnetic field. Furthermore, the geometry is also found to favor the formation of ordered normal/superconducting domains along the length of the helix, exhibiting a matching effect comparable to those induced by thickness modulation in a thin film (discussed below in Section 3.5). This effect is observed through a transition to the superconducting state in a series of discrete steps, ascribed to the occurrence of phase-slip phenomena, and accounted for by Ginzburg–Landau theory-based numerical simulations.

With FIBID W-C being superconducting, it is only logical to consider whether the growth using an FEB instead of an FIB would yield a material with comparable superconducting properties. Early reports in the matter indicated the lack of superconducting behavior down to 2 K [77], deeming this approach unfeasible. Notwithstanding, a single report by Sengupta et al. [78] presented the fabrication of superconducting W-C FEBID nanowires with a composition W:C:O ratio of 50:40:10 and a critical temperature of 2 K.

More recently, Blom et al. [79] reported the growth of a superconducting W-C material by using comparatively higher FEB currents, with a critical temperature of 5.7 K. Although the usage of a higher beam current compromises the resolution of the technique, this approach allows one to fabricate superconducting nanodevices without the main drawbacks of Ga${}^{+}$ FIBID, and has been exploited to fabricate Josephson junctions.

#### 2.2.2. Niobium

Niobium, a classical example of an element present in superconducting materials, both in bulk form and constituting alloys, can be patterned by FIBID/FEBID of the Nb(NMe${}_{2}$)${}_{3}$(N-t-Bu) precursor [80].

Porrati et al. [13], in 2019, reported the fabrication of Nb-based nanowires by Ga${}^{+}$ FIBID. As characterized by EDS, the deposits exhibited an atomic Nb content of 28.7%, with a constituent Nb:C:Ga:N ratio of 28.7:42.9:15.5:12.9 in terms of atomic percentage. TEM analysis indicate that the microstructure of the material is that of an array of partially touching crystallites with sizes in the 15 nm to 20 nm range. Electron beam irradiation of the FIBID nanowires was also shown to improve the quality of the nanocrystalline structures.

In-plane nanowires exhibited a room-temperature resistivity $\rho_{N}$ of 550 μΩ·cm and a superconducting transition at $T_{c}=5.0$ K. These figures were improved on by irradiating the samples with the electron beam, lowering the room-temperature resistivity to 520 μΩ·cm and raising $T_{c}$ to 5.4 K.

More significant differences were found in the study of freestanding NbC nanowires also prepared by Ga${}^{+}$ FIBID: with $\rho_{N}=380\;$μΩ·cm and $T_{c}=8.1$ K, further raised to 11.4 K by hypothesized electromigration (i.e., inducing graphitization on the material by making a current flow through the nanodevice for an extended period of time [81]). With the onset of the transition approaching the value of $T_{c}$ in bulk NbC, the authors attribute the origin of superconductivity in this material to the nano-crystalline microstructure. In that same study, Porrati et al. reported that NbC nanowires grown by FEBID of Nb(NMe${}_{2}$)${}_{3}$(N-t-Bu) show insulating behavior at low temperatures.

#### 2.2.3. Molybdenum

A molybdenum-based superconducting material may be obtained by both FIBID and FEBID of the Mo(CO)${}_{6}$ precursor. The FIBID approach was first reported by Weirich et al. [82] in 2014, making use of a focused beam of Ga${}^{+}$ ions.

Using a standard FIBID configuration, and setting the acceleration voltage to 30 kV and the ion beam current to 10 pA, the resulting material exhibits room-temperature resistivity in the 300 μΩ·cm to 600 μΩ·cm range, comparable to that obtained in the previous materials. With the optimized growth parameters, the component ratio Mo:C:Ga:O is 41.5:25.7:26.1:6.7 in terms of atomic composition. The authors remark that these resistivity values indicate the proximity of the material to a disorder-induced metal-insulator transition [83]. Similarly to the behavior observed in FIBID W-C, the resistivity of this material increases as the temperature is reduced, and then sharply drops to zero when the critical temperature is reached, found in the 2.7 K to 3.8 K range. The largest values of $T_{c}$ are found in those materials exhibiting lower Ga and C atomic content. In addition, the critical magnetic field at 0 K is extrapolated to 2.5 T to 4.5 T.

On the other hand, as demonstrated by Makise et al. [84] in 2014, superconducting MoC may also be obtained by FEBID of a mixtures of the Mo(CO)${}_{6}$ precursor with water vapor, H${}_{2}$O. As reported, increasing the partial pressure of H${}_{2}$O during the deposition process results in a reduced amount of oxygen being present in the deposit, down to a near-complete suppression of O presence at a threshold value of the partial pressure ratio of the two gases. With a minimal influence of the hydrogen from the water vapor, the final contents of Mo and C were found to be 70% and 30%, respectively.

As evidenced by TEM analyses, the as-grown MoC material is microstructurally amorphous, yet its crystallization may be induced by post-annealing it. Notwithstanding, only the amorphous material shows superconducting behavior: showing a semiconductor-like resistivity-temperature dependence (increasing with decreasing temperature down to 10 K, then decreasing down to the critical temperature) and a superconducting transition at 7.2 K.

#### 2.2.4. Other Precursors

Two last examples of superconducting materials obtained by FIBID and FEBID are carbon and lead-based nanostructures.

Concerning the former, Dhakal et al. [85] reported in 2010 the fabrication of carbon-based superconducting nanostructures by Ga${}^{+}$ FIBID of phenanthrene (C${}_{14}$H${}_{10}$). Grown using an acceleration voltage of 30 kV and an ion beam current of 100 pA, the nanowires exhibited an incomplete transition to the superconducting state near 7.0 K, and an upper critical field of 8.8 T. TEM analysis indicated that the material lacked any crystalline structure, and that the C:O:Ga ratio was of 34:38:26 in terms of atomic composition.

Lead-based superconducting deposits may be obtained by FEBID of the tetraethyllead ((CH${}_{3}$CH${}_{2}$)${}_{4}$Pb) precursor material, as reported by Winhold et al. [86] in 2014. The resulting material exhibits a Pb:C:O ratio in the range of 40.1:36.5:23.4 to 45.8:31.2:23, largely independent of the voltage and current employed to grow it. Samples with Pb content above 43% exhibit metallic behavior, with decreasing resistivity from a room-temperature value near 166 μΩ·cm, down to a transition to the superconducting state near 7.2 K.

A more detailed analysis, based on a fit to the Ginzburg–Landau equations of superconductivity, revealed that in some samples, the temperature dependence of the material could not be explained by a single superconducting transition, but rather by the co-existence of two superconducting phases. The first phase has an associated critical temperature of 7.2 K and is very susceptible to the external magnetic field, which promptly suppresses it. After that magnetic field-induced suppression, a second phase with a lower $T_{c}$ of 6.6 K, and a significantly enhanced associated zero-temperature critical field of 9.9 T, dominates. The corresponding coherence length for this lower $T_{c}$ phase is 5.75 nm; while the critical field and coherence length for the higher $T_{c}$ phase are 0.20 T and ξ=40.23 nm (both phases appear separated with a slash in Table 1).

The authors ascribe the appearance of these distinct superconducting phases to the incorporation of C to the nanostructure as a side effect of the FEBID procedure. Since the Pb-C bond in the precursor molecule is weak enough for the FEB to decompose effectively, the Pb content in the deposited nanostructures is sufficiently high for them to exhibit a $T_{c}$ comparable to that of bulk lead. Such a higher $T_{c}$ is only observed at low magnetic fields, and when the presence of this phase in the nanostructure is significant enough to constitute a percolating path. The lower-$T_{c}$ phase, richer in C, dominates after applying a magnetic field that suppresses the superconductivity of the higher-$T_{c}$ phase. The preferential appearance of one of the phases over the others is strongly influenced by the deposition parameters.

## 3. Applications

As previously discussed, the main two virtues of FIBID and FEBID reside in its single-step nature, and in its patterning flexibility. Concerning the former, dropping the requirement to use a resist that may contaminate or otherwise hamper the process provides with a significant advantage over other techniques. In addition, the technique allows for great adjustability in both designing the pattern and the substrate for the deposit to be grown. In combination, these two factors allow for complex patterning in conventional and unconventional substrates, making the technique a powerful tool.

The W(CO)${}_{6}$ precursor is undoubtedly the most reported and utilized material for the nanopatterning of superconducting devices using focused beams. Thanks to its commercial availability and the high reproducibility exhibited in the growth, its deposition is conveniently achieved with ease. Moreover, the superconducting parameters of the deposited material make for an experimentally accessible investigation, which further settles W-C as the most popular option for nanopatterning of superconductors using FIBID. Thus, W-C has been the material of choice for most of the reported applications in superconductivity, which exploit it in different ways.

Materials grown by FIBID and FEBID represent an inciting object of study by themselves, exhibiting the unique and defining characteristics imposed by the nature of the deposition process. In this section, several remarkable examples of the application of such materials (mostly centered in W-C) are presented.

### 3.1. Superconducting Proximity Effect

The possibility of controlling the superconducting properties at the nanoscale, offered by superconducting materials grown by FEBID or FIBID, paves the way for the investigation of physical effects that occur at interfaces at the nanoscopic scale. A relevant example is the superconducting proximity effect [87,88]. It occurs at the interface between a normal metal (N) and a superconductor (S) when both materials are brought into electrical contact with each other. Superconducting properties could be induced in the normal material, while degrading in the superconducting material forming the interface. Superconducting proximity effect takes place through Andreev reflection at the interface, which involves an electron reaching the superconductor—normal interface from the normal side, being retroreflected as a hole and accordingly creating a Cooper pair in the superconductor [89]. It is remarkable that this process makes it possible that a current flows without dissipation through a normal metal if two superconductors are close enough to each other and in electrical contact to the normal metal. When the normal metal is replaced by a ferromagnetic (F) one, if the superconductor forming the F–S contact is a conventional superconducting material, it results in conventional superconducting proximity effect in which there is a strong pair-breaking effect that causes an exponential decay of the superconductivity in the F side [90]. However, if the superconductor forming the F–S interface presents odd triplet superconductivity, long-range proximity effect arises because superconducting correlations can extend to the F side over much longer distances [91]. The high resolution and versatility offered by FEBID and FIBID techniques makes them very useful techniques for investigations of the superconducting proximity effect. The most used material in this type of studies is W-C grown by Ga${}^{+}$ FIBID due to its remarkably high upper critical field that allows one to investigate this interfacial effect in a wide range of magnetic field values.

One of the first experiments of proximity-induced superconductivity by using superconducting nanoelectrodes fabricated by FEBID/FIBID was reported by Kasumov et al. [92]. The controlled fabrication of nanometer-size gaps between micron-long suspended W-C nanoelectrodes deposited by Ga${}^{+}$ FIBID and the deposition of Gd metafullerenes molecules in the gaps enabled the study of proximity-induced superconductivity in these molecules, finding that the proximity effect is very sensitive to the magnetic state of the molecules.

The study of the electronic transport properties of few-layer graphene connected to superconducting electrodes was reported by the same group in 2007 [93]. By mechanically exfoliating graphite, a sheet of few-layer graphene is connected to two W-C superconducting electrodes, separated by 2.5 μm and grown by Ga${}^{+}$ FIBID. The superconducting proximity effect in the few-layer-graphene foil occurs at around 1 K and the different peaks observed in the differential resistance measured below this temperature are interpreted as the signature of incoherent multiple Andreev reflections in the W-C—few-layer graphene—W-C devices.

In 2011, the same group also reported the fabrication of N—S rings composed by two superconducting electrodes on both sides of a gold wire [94]. Superconducting W-C nanoelectrodes were deposited by Ga${}^{+}$ FIBID. They measured the high frequency linear response of these N—S rings when inductively coupled to a multimode superconducting resonator. The dynamics of Andreev states was studied, showing that at low temperatures the response is non-dissipative but there is a strong additional dissipative component at higher temperatures (around 1 K).

Proximity-induced superconductivity in bismuth nanowires has also been studied by H. Bouchiat’s group. They first reported the transport properties of micrometer-long 90-nm-wide bismuth nanowires connected to W-C superconducting electrodes grown by Ga${}^{+}$ FIBID [95]. Taking advantage of the high upper critical field of Ga${}^{+}$ FIBID W-C nanodeposits, they were able to measure the persistence of supercurrent in magnetic fields as high as 11 T in the Bi nanowires. They found periodic oscillations of the critical current occurring up to high magnetic fields, the period and decay of these oscillations indicate that the interferences occur between strongly confined 1D channels, possibly located at the edges of specific facets of the nanowires. Few years later they reported the investigation of superconducting proximity effect in micrometer-long 30-nm-wide 200-nm-thick monocrystalline bismuth nanowires, connected to two superconducting Ga${}^{+}$FIBID W-C electrodes [96]. They measured the relation between the Josephson current flowing through the bismuth nanowire and the superconducting phase difference at its ends, finding that the contribution of the two ballistic edge states of the nanowire outweighs that of the diffusive channels on the non-topological surfaces. In addition to being ballistic rather than diffusive, they found that topological edge states are better coupled to superconducting electrodes due to their spin-momentum locking.

**Figure 4:**
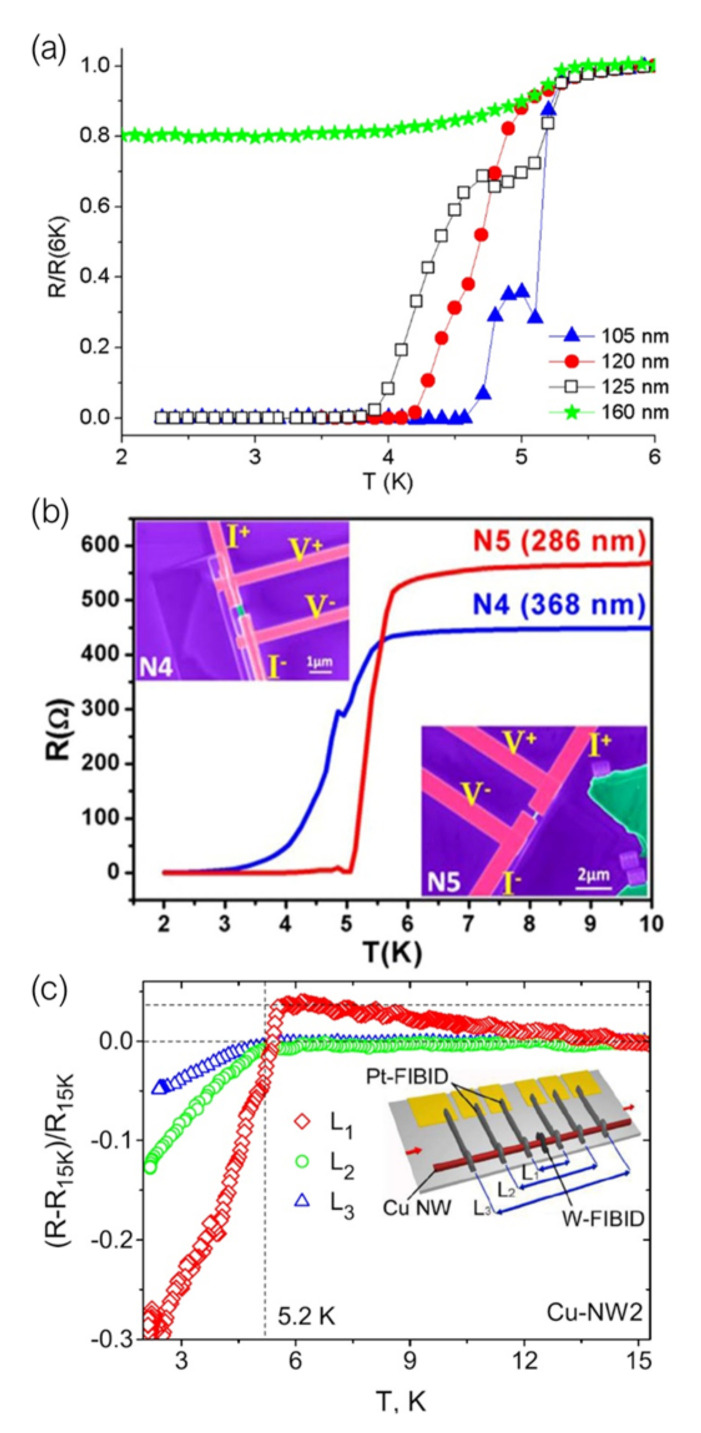
(**a**) Resistance, normalized at its value at 6 K, as a function of temperature for W-C—bismuth nanostripes—W-C devices with inner probe distances of 105 nm, 120 nm, 125 nm, and 160 nm. Adapted from [97], with permissions from IOP Publishing. (**b**) Resistance as a function of temperature for W-C—Bi${}_{2}$Se${}_{3}$—W-C devices N4 and N5, with junction lengths 368 nm and 286 nm, respectively. Insets show false-colored SEM micrographs of the devices with Ga${}^{+}$FIBID W-C superconducting electrodes in pink and Bi${}_{2}$Se${}_{3}$ nanowires in green. Reprinted from [98], under CC BY license. (**c**) Relative change in resistance, normalized at its value at 15 K for single-crystal Cu nanowires with an inner Ga${}^{+}$FIBID W-C superconducting inducer electrode. Resistance measurements were performed by probing different lengths between 2 μm and 12 μm. The inset shows the scheme of the electrical contacts used for the resistance measurements. Reprinted from [99], with permissions from AIP Publishing.

Another example of induced superconductivity in bismuth by using superconducting electrodes grown by Ga${}^{+}$ FIBID was reported by our group in 2017 [97]. The possibility of inducing superconductivity in bismuth nanostripes through electrical contact with W-C nanodeposits grown by Ga${}^{+}$ FIBID was investigated, finding, as illustrated in Figure 4a, that bismuth nanostripes with inner probe distance shorter than 125 nm become superconductor at temperatures above 2 K due to proximity-induced superconductivity. From the study of the electrical transport properties of these bismuth-nanostripes-based S–N–S devices, the superconducting proximity length in bismuth was estimated to be around 120 nm at very low temperatures.

The interest in studying proximity-induced superconductivity on strong spin-orbit coupling materials, such as bismuth, relies on the possibility of designing and fabricating devices based on Majorana excitations occurring in these systems for fault-tolerant quantum computation. One of the first studies of proximity-induced superconductivity in topological insulators, very interesting for the possibility of the formation of Majorana fermionic excitations at the interface of a topological insulator with a conventional superconductor, was performed by Zhang and co-workers [100]. The study reports the transport properties of Bi${}_{2}$Se${}_{3}$ nanoribbons interfaced with superconducting W-C contacts deposited by Ga${}^{+}$ FIBID. They observed multiple Andreev reflection for channel lengths much longer than the inelastic and diffusive thermal lengths, which suggests that the superconducting proximity effect couples preferentially to a ballistic surface conduction channel in topological insulators, even when diffusive bulk conduction coexists. Moreover, they observed magnetoresistance oscillations when the nanoribbons were place in a perpendicular magnetic field that can be explained using a Weber blockade model in which the perpendicular magnetic field controls the number of Pearl vortices in the superconducting nanoribbon.

Supercurrent studies on nanowires for its implementation in quantum computation devices was taken a step further by Bhattacharyya et al. [98]. They fabricated topological insulator, Bi${}_{2}$Se${}_{3}$, nanowires proximity-coupled to W-C electrodes grown by Ga${}^{+}$ FIBID. A sharp superconducting transition was observed for the shortest Bi${}_{2}$Se${}_{3}$ nanowires (Figure 4b) and the dominant role of topological surface states in propagating the long-range proximity induced superconductivity was demonstrated.

Proximity-induced superconductivity in gold nanowires connected to two superconducting Ga${}^{+}$FIBID W-C electrodes as a function of the length of the nanowire has also been studied by M. H. W. Chan’s group [101]. They found that short gold nanowires exhibit a sharp superconducting transition, long nanowires show nonzero resistance, whereas at intermediate lengths, there are two sharp transitions, indicating that the normal and superconducting regions are separated by a minigap state. This state originates from a coexistence of proximity-induced superconductivity with a normal region near the centre of the wire.

Our group reported for the first time the possibility of combining superconducting nanostructures grown by FIBID with magnetic nanostructures grown by FEBID to fabricate functional S–F devices [102,103]. Planar nanocontacts between a ferromagnetic Co nanodeposit grown by FEBID and a superconducting W-C nanodeposit grown by Ga${}^{+}$FIBID were created. The magnetic field dependence of the conductance in these S–F nanocontacts was studied, which allowed us to extract both, the spin polarization of the Co-based nanodeposit and the magnetic field dependence of the superconducting gap of W-C nanodeposits.

A similar attempt of combining FIBID-grown superconducting nanostructures with FEBID-grown magnetic nanostructures was reported by Sharma et al. [104]. They fabricated nanocontacts formed by Co-based nanodeposits grown by FEBID and W-C nanodeposits grown by Ga${}^{+}$ FIBID. They observed multi-channel electron transport, revealed by the presence of multiple shoulders in Andreev reflection measurements and explained in terms of the properties of the W-C superconducting nanodeposit and the specific structural properties at the interface.

Interplay between superconductivity and ferromagnetism when using Ga${}^{+}$FIBID W-C superconducting electrodes has been investigated by M. H. W. Chan’s group. They sandwiched ferromagnetic Co and Ni nanowires between two W-C electrodes grown by Ga${}^{+}$ FIBID [105]. They found that both ferromagnetic Co and Ni nanowires exhibit proximity-induced superconductivity when connected to superconducting W-C electrodes over a spatial extent of the order of 500 nm, similar to the value they had found in paramagnetic gold nanowires [101]. Co and Ni nanowires that do not reach zero resistance at low temperature, show and unexpected resistance peak immediately before the onset of superconductivity, not present in their measurements of gold nanowires.

Transport properties in S–F devices in which the superconducting electrode is deposited by means of FIBID have been extensively studied by M. Huth’s group [99,106,107]. They first reported the study of the superconducting proximity effect in polycrystalline Co nanowires in contact with a W-C superconducting inducer floating electrode [106]. A long-range superconducting proximity effect was observed in these Co nanowires, with a proximity length of around 1 μm at 2.4 K. They later published the study of proximity-induced superconductivity in single crystal Cu nanowires and FEBID-grown Co nanowires [99]. In this case they used the same inner W-C superconducting inducer electrode grown by Ga${}^{+}$ FIBID but they added two extra pairs of Pt-based voltage leads to probe the proximity effect over three different distances, as shown in Figure 4c. The proximity length in the single crystal Cu nanowires was estimated to be 1.6 μm at 2.4 K, while proximity-induced superconductivity in nanogranular Co nanowires grown by FEBID was found to be strongly suppressed due to the dominating Cooper pair scattering caused by its intrinsic microstructure.

### 3.2. Vortex Dynamics

#### 3.2.1. Vortex Observation in W-C Deposits

As aforementioned, the W-C deposits fabricated by Ga${}^{+}$ FIBID exhibit type-II superconductivity, with a lattice of superconducting vortices populating the material when it is subject to an externally applied magnetic field. As such, the material also provides with an excellent playground for the investigation of several physical phenomena related to the detection, motion and behavior of superconducting vortices.

**Figure 5:**
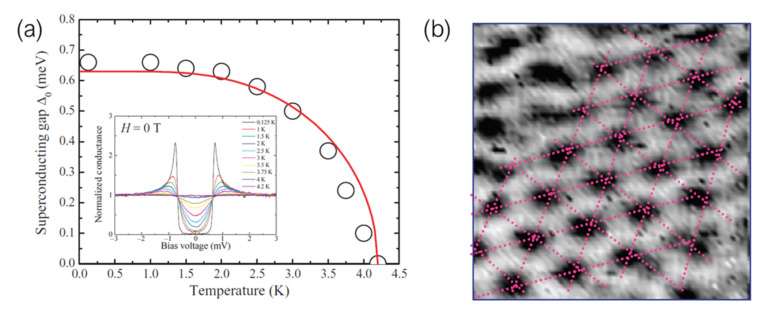
(**a**) Temperature dependence of the superconducting gap, as probed by STS. Inset shows individual spectra taken at different temperatures. Reprinted from [73] (https://doi.org/10.1088/1367-2630/10/9/093005, accessed on 15 March 2022). *©* IOP Publishing Ltd and Deutsche Physikalische Gesellschaft. Reproduced by permission of IOP Publishing. All rights reserved. (**b**) Abrikosov vortex lattice in a W-C thin film at 1.2 K and 2 T, as imaged by STS, with each dark region corresponding to an individual vortex. Pink lines outline the hexagonal symmetry of the lattice. Lateral image size: 220 nm. Reprinted from [110], with permissions from Springer Nature.

One first approach to explore vortex physics is by means of *Scanning Tunneling Microscopy/Spectroscopy* (STM/STS) [108], a scanning probe technique that makes use of an atomically sharp tip to sequentially probe the density of states of a sample by measuring the changes and dependence of the tunneling current that is established between the two when they are counter-biased. The electronic structure of a superconductor is unmistakably defined by the presence of a *superconducting gap*. In the mixed state, the superconducting bulk does exhibit such a gap, but the normal cores of any present vortices do not, which allows for the retrieval of STS images where vortices can be easily visualized (Figure 5).

Guillamón et al. have extensively investigated the superconducting properties and vortex dynamics of W-C thin films nanopatterned by Ga${}^{+}$ FIBID. Such films proved to be a system with a level of surface homogeneity appropriate for STM experiments. Measurements indicate a good fit to the *s*-wave BCS model of superconductivity, with a value of the superconducting gap of 0.66 meV, and a subsequent gap dependence with temperature also in agreement with that theory. Superconducting vortices populate the sample above a lower critical magnetic field of 0.3 T, forming arrangements of hexagonally distributed vortices between surface deffects [73,109].

A remarkable investigation performed on such W-C thin films is that of the observation of the temperature-induced melting of the vortex lattice. The STS setup in combination with the homogeneous W-C material enables for direct, dynamical microscopic observation of the evolution of the hexagonal lattice. At a fixed value of magnetic field, the following several phases can be observed in the order of increasing temperature: a (relatively) disordered vortex array, with the distribution mainly determined by local pinning sites; a more ordered lattice, with the re-ordering of the vortices driven by thermal depinning; a *hexatic phase* after the onset of melting, identified by the appearance of free dislocations in the film, and followed by smectic-like vortex arrangements; an isotropic liquid of thermally-excited vortices that are able to move faster than the scan speed of the instrument; and finally, the normal state [110].

Similarly, the direct evolution of the lattice was also monitored as a function of an increasing magnetic field. While the “initial” vortex positions are determined by the surface roughness, which translates to linear extended pinning centers, it is the distribution of the different pinning centers which determines the direction of motion. Observing the evolution of the lattice in small steps of magnetic field, *vortex avalanches* are observed, in which several vortices (that form a “bundle”) quickly switch between different arrangements, all at a time [111].

The technique was also exploited to investigate the correlation effects that arise between the lattice and an artificially induced pinning potential, in the form of a periodic thickness modulation (which will also be later discussed as a means for vortex immobilization, in Section 3.5). With the coupling strength between the two depending on the magnetic field, two distinct phases are once again observed as this parameter is sequentially increased: a vortex lattice that is “locked” to the film corrugation (or “commensurate” with it), and a floating 2D “solid”, with an arrangement independent of the pinning potential [112].

Overall, the technique-sample combination provided by STM and Ga${}^{+}$ FIBID W-C gives rise to a convenient scenario for the microscopic investigation of superconductivity phenomena, not only limited to vortex dynamics, but also potentially including the detection of nanoscale Bitter effect [113] and Yu-Shiba-Rusinov states [114]. At the time of writing, such studies are still to be carried out.

#### 3.2.2. Non-Local Vortex Transport

Injecting a current in a type-II superconductor material in the mixed state induces a force in any present vortices by electrostatic interaction between the charges in their screening circular currents and the charge carriers of the injected current. The normal cores dissipate energy as they drift along the superconducting bulk, yielding a finite resistance to the superconducting material [3,115]. Energy dissipation is generally counter-productive in superconductivity applications, and as such, vortex motion within a type-II superconductor is usually regarded an unwanted effect, and immobilization of the vortices is a desirable goal [116,117].

Contrary to this trend, vortex motion can also be a productive and interesting goal by itself, should it be considered the actual object of study rather than a nuisance that hinders superconductivity. The possibility of transporting vortices to areas depleted of current was first presented in 1965 through Giaever’s sandwich-like Sn–SiO${}_{2}$–Sn device [118]. By injecting a current through one of the (type-II superconducting) Sn films, a finite resistance was detected in the other, the two films being insulated one from the other by the intermediary SiO${}_{2}$ layer. In virtue of the stiffness of the Abrikosov lattice, the repulsive force between neighboring vortices resulted in net motion of the whole array, promoted at the region where the driving current was injected, but with this repulsion extending through the insulating barrier and reaching the other superconducting film. The transport of vortices to areas depleted of current, and the subsequent detection of the finite voltages they yield as they move is referred to as *non-local transport*, relating to the fact that the motion and its associated resistivity extend beyond the vicinity of the driving current injection point.

**Figure 6:**
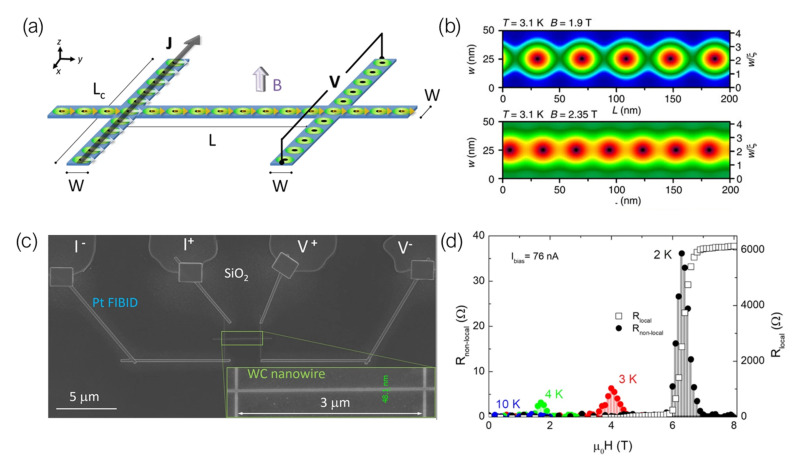
(**a**) Schematic representation of the non-local configuration for vortex transport measurements. (**b**) Ginzburg–Landau theory-based numerical simulations of the distribution of the superconducting parameter in a 50 nm-wide nanowire, evidencing the single-row distribution of vortices along the axis. Reprinted from [65], with permissions from Springer Nature. (**c**) SEM image of a superconducting W-C nanowire fabricated by Ga${}^{+}$ FIBID, showing the supporting contacts placed for non-local voltage measurements. (**d**) Non-local magneto-resistance curves of the previous nanowire, with the characteristic non-local resistance peaks appearing near the transition at each temperature. Figures (**a**,**c**,**d**), reprinted from [121], under CC BY license.

In-plane non-local vortex transport in nanostructures was first investigated by Grigorieva et al. in a MoGe device patterned as a nano/micro-wire with two leads perpendicularly placed to its longitudinal axis [119]. Injecting a driving current through one of these leads results in a force being exerted on vortices present therein, which in turn push other neighboring vortices in the lattice. With the momentum transfer being maintained throughout the whole length of the nanostructure, vortices passing through the opposite lead, depleted of current, yield a finite voltage as they pass through (Figure 6a).

This effect has been further explored in superconducting devices fabricated by Ga${}^{+}$ and He${}^{+}$ FIBID of W(CO)${}_{6}$. As reported by Córdoba et al., by patterning a nanowire whose width is comparable to the coherence length of the W-C material, the energetically favorable arrangement of the Abrikosov lattice is that of a single row of vortices along the long axis of the nanowire, confined between potential barriers at the edges [65].

As such, the non-local transport in devices with a channel width in the order of 50 nm is constrained to a single row of vortices. It has been experimentally found that such a geometry yields a more efficient vortex transfer—while transport experiments in 200 nm-wide nanowires yield an equivalent non-local resistance in the order of mΩ (estimated using Ohm’s law, V=IR), by reducing the width of the channel, the non-local signal is enhanced to the order of Ω [120].

This enhancement in the effectiveness of vortex transport has been confirmed by both retrieving significantly high values of non-local resistance associated to long-range transport: up to 36 Ω in a 3 μm-long nanostructure, and 9 Ω in a 10.25 μm-long one [121]. Numerical simulations based on the Ginzburg–Landau theory of superconductivity indicate that the effect is in principle able to persist among distances much greater than the lattice parameter, which further reinforces the applicability of this technique-material pairing for the fabrication of vortex-based nanocircuits. Similar experiments have also been carried out in nanowires fabricated by He${}^{+}$ FIBID of W(CO)${}_{6}$, yielding equivalent results to those retrieved when Ga${}^{+}$ FIBID was used [75].

The potential of vortex-based nanocircuits lies in the quantized nature of the vortices. Each of them encloses a precisely-defined and known amount of magnetic flux which depends on physical constants only, and is therefore not affected by the experiment conditions. More general examples that showcase the prospective applications, to date still not demonstrated in nanodevices fabricated by FIBID, are their consideration as quantized information carriers [122] (this example being the most tailored to the described non-local transport), the tuning of the Josepshon effect at a junction by an optically-driven vortex [123], and the possibility of controlling their generation in superconducting nanotubes [124].

While the MoGe samples from the original experiments of Grigorieva et al. were fabricated by electron beam lithography, but at the time of writing, the highest reported absolute values of non-local voltage have been detected in narrow nanowires fabricated by FIBID, which reassures its viability for this particular purpose. Thus, the patterning flexibility of FIBID and FEBID is most welcome in the design of such vortex-based nanocircuits, and as such, it represents the most promising approach towards their further potential applicability in the examples outlined above.

#### 3.2.3. Ultra-Fast Vortex Transport

Vortex dynamics have also been investigated by Dobrovolskiy et al. in Nb-C microstripes fabricated by Ga${}^{+}$ FIBID of the Nb(NMe${}_{2}$)${}_{3}$(N-*t*-Bu) precursor [125]. Specifically, the authors focus on *ultra-fast* velocities (≳5 km/s), for these values are expected to shed light on interesting physics as the speed of the vortices exceeds that of other excitations in the system [126,127].

Ga${}^{+}$ FIBID-grown NbC does exhibit strong edge barriers, a value of the critical current close to its depairing value, and a fast quasiparticle relaxation time, all of which are highly convenient factors in ultra-fast vortex dynamics investigations. Paramountly, nanopatterning this material by FIBID yields near-perfect edge-barriers that restrict and order vortex motion at high currents, allowing for the investigation of the aforementioned ultra-fast regime, which would otherwise not be possible as the flux-flow instability phenomenon takes over the superconducting state [128]. The nanowires are able to sustain ultra-fast vortices that nucleate at one of the edges of the microstrip, and driven by the injected current, transversally cross the strip through highly resistive “vortex rivers” [129,130]. These results indicate the potential applicability of FIBID NbC for the fabrication of single-photon detectors (discussed below in Section 3.5).

#### 3.2.4. Emergent Phenomena in 3D Nanostructures

Within the framework of the Ginzburg–Landau theory, superconductivity is described by means of an *order parameter*, whose absolute value is physically related to the presence or absence of the superconducting gap at a given location [3]. Superconductors are characterized by a strong interplay between the physical geometry of the sample and the topological properties of this order parameter, which results in the appearance of interesting phenomena when the superconductor is nanostructured with geometries different to conventional in-plane arrangements [131,132].

Vortices behave as defects in the topology of the superconducting parameter and are therefore also sensitive to this interplay. Geometrically breaking the symmetry of the superconductor in the presence of an externally applied magnetic field gives rise to strong inhomogeneities in the field components and to the appearance of complex patterns in the Meissner screening currents that arise therein, which eventually lead to the occurrence of vortex-related effects that are simply absent in “simpler” geometries. Recent reviews on the matter may be found in [131,132].

Three dimensional (3D) nanopatterning of superconducting nanostructures represents one plausible approach towards the exploration of such geometry-topology interplay effects, and FIBID and FEBID exhibit the potential for it. The aforementioned 3D W-C nanohelices patterned by He${}^{+}$ FIBID [30] represent one notable example, whose characterization evidences the appearance of complex vortex and phase slip patterns that are not found in 2D W-C nanostructures. These features exhibit the potential to utilize 3D-grown He${}^{+}$ FIBID W-C in the fabrication of metamaterials [133] and for the study of commensurability effects [134]. Together with the already-exhibited capability of FEBID in the fabrication of complex 3D magnetic nanostructures [135], both FIBID and FEBID hold great potential towards the nano-printing of intriguing 3D nanostructures, bound to undergo interesting development and application in the coming years.

### 3.3. Electric Field-Induced Critical Current Modulation

Tuning the resistivity of a semiconductor material by applying an electric field in its vicinity is one of the most important founding stones of modern circuitry, with the design of gate voltage-modulated transistors being firmly rooted in this principle. Modifying the electrical properties of a metallic material in a similar manner is, in principle, not feasible, since the large density of free charge carriers in metals induces an effective screening of the electric field as soon as it is applied, nullifying its influence in the bulk.

Very recently, however, the research community in superconductivity has taken a keen interest in the possibility of achieving this kind of control in superconducting nanostructures. It has been experimentally found that the application of an external electric field in close proximity to a superconducting channel can quench the non-dissipating state, thus enabling for an externally controlled switching of the resistivity of the material via the application of a gate voltage. Such an effect has been observed in titanium [136,137], aluminium [138], vanadium [139], and niobium [140] nanodevices, fabricated by various lithography approaches (other than FIBID/FEBID).

**Figure 7:**
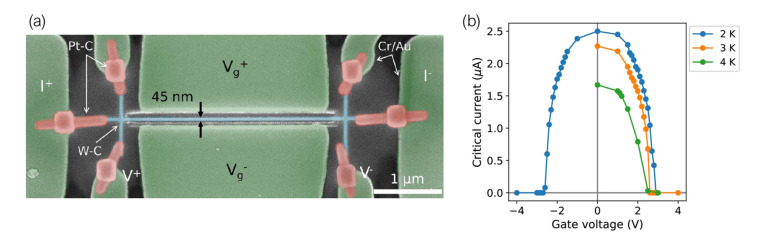
(**a**) Artificially colored SEM image of a W-C nanowire placed between two gate electrodes. (**b**) Critical current dependence of the nanowire with the side-gate voltage. Reprinted from [141], under CC BY license.

The effect has also been observed in W-C nanowires fabricated by Ga${}^{+}$ FIBID [141]. The application of an increasing side-gate voltage to a 2.7 μm-long, 45 nm-wide W-C nanowire is found to progressively reduce its critical current, down to a full suppression of the superconducting state at a comparatively low voltage of 3 V (Figure 7). These experiments have added FIBID to the list of nanopatterning techniques suitable for the fabrication of devices in which such a phenomenon may be observed.

A complete and fully accepted microscopical justification for the occurrence of the phenomenon is, at the time of writing, still lacking. Reports on the observation of the effect have stirred up discussion on the matter with mainly two proposed (and competing) hypotheses: an electrostatic-field effect akin to that characteristic to semiconductors [142,143,144,145], and the injection of energetic electrons from the gate electrodes, which trigger the formation of quasiparticles which in turn are responsible for the transition to the normal state [146,147,148]. The more recent experiments trend towards highly tailored approaches to rule out or confirm some of the hypotheses, for which the FIBID capability of at-will patterning might prove of use in the near future.

### 3.4. Fabrication of SQUIDs

The aforementioned proximity effect occurring in superconductor-normal metal-superconductor (SNS) heterostructures represents a special case of a *Josephson Junction* (JJ) [149]. First described in a Superconductor-Insulator-Superconductor (SIS) heterostructure, a phase sensitive superconducting tunnel current is able to pass such an insulating barrier. The same effect was later found in SNS junctions [150] as well as in constrictions in the superconductor, i.e., Dayem Bridges (DBs) [151]. An intriguing application of the Josephson effect is its usage in *Superconducting Quantum Interference Devices* (SQUIDs), i.e., superconducting magnetic field sensors with a high magnetic field sensitivity.

SQUIDs consist of a superconducting ring, interrupted by two JJs in parallel. An injected current splits into two Josephson currents running through either of the JJs. The flux of an external magnetic field is repelled from the superconductor and focused into the ring in integer multiples of the magnetic flux quantum, $\Phi_{0}$. The flux quantization is ensured by the induction of a circular current in the SQUID loop, supporting or opposing the flux to an integer multiple of $\Phi_{0}$. This results in a periodic modulation of the critical current, $I_{c}$ in dependence of the magnetic flux with a period of $\Phi_{0}$. In turn, the modulation of the critical current results in a sinusoidal voltage drop across the structure upon injection of a constant bias current $I_{b}∼I_{c}$, thus forming a sensitive flux-voltage-transducer. For a comprehensive review of the working principle of a SQUID please refer to [152].

It was long found that the sensitivity scales reciprocally with the inductance, which can in turn be reduced by reducing the size of these devices, which lead to the development of various techniques for the fabrication of *nanoSQUIDs* [153,154,155,156]. A comprehensive review on the recent advancements in nanoSQUIDs can be found in [157].

**Figure 8:**
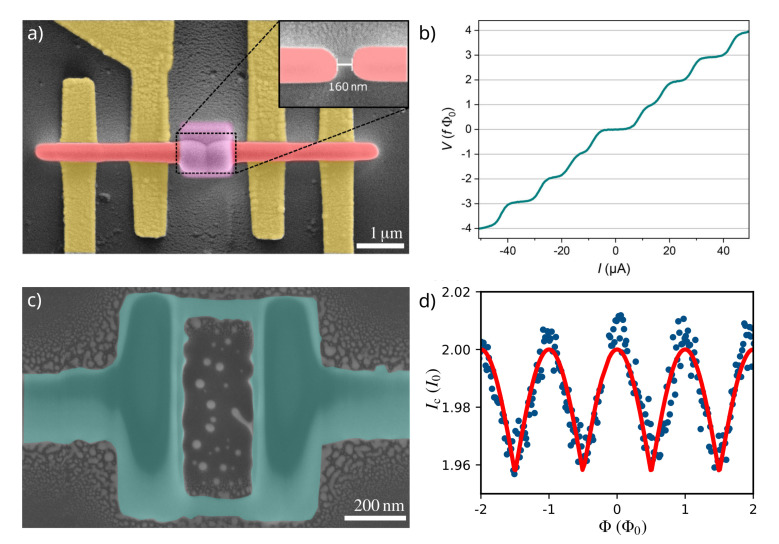
(**a**) SEM image of a W-C SNS-JJ fabricated by a single-step, direct write FEBID process. The superconducting leads (red) are deposited with a high electron beam current whereas the normal conducting part (purple) is deposited with a low electron beam current. Yellow represents the Au leads. (**b**) The current voltage characteristics of the SNS-JJ exhibiting the expected Shapiro steps. Reprinted from [79] with permissions from ACS Publications (https://pubs.acs.org/doi/10.1021/acsnano.0c03656, accessed on 15 March 2022). Further permissions related to the material excerpted should be directed to the ACS. (**c**) SEM image of a W-C DB-nanoSQUID fabricated by Ga${}^{+}$ FIBID and (**d**) the corresponding modulation of the critical current $I_{c}$. Reprinted from [158].

Recently, Blom et al. reported the fabrication of SNS-JJs with a junction length of 160 nm from W-C by means of FEBID (Figure 8a) [79]. The structures exhibit Shapiro steps in the current versus voltage characteristics upon microwave irradiation of 6.4 GHz (Figure 8b) as well as an $I_{c}\left(B\right)$ dependence resembling a Fraunhofer diffraction pattern, as expected for a single JJ. In the structure, the superconducting part is deposited using a high electron beam current, whereas the normal conducting region results from irradiation with a low electron beam current. Contrary to conventional methods, e.g., Electron Beam Lithography (EBL), the entire structure is directly written in a single step procedure.

Alternatively, the JJs can be fabricated by FIBID. Recently, our group demonstrated the single-step fabrication of a SQUID by connecting two large W-C pads of 50 nm thickness with two W-C nanowires (50 nm in width, and 300nm in length) fabricated by Ga${}^{+}$ FIBID [158]. The nanowires form DBs, resulting in two parallel JJs connecting the pads (Figure 8c). The resulting structure exhibits the expected oscillation of the critical current as well as the voltage modulation at constant $I_{b}$ (Figure 8d). The resulting W-C SQUIDs suffer from a low modulation depth, i.e., a reduced difference between the minimum and the maximum of $I_{c}$, in comparison with SQUIDs fabricated from conventional superconductors by means of EBL. The low modulation depth is due to the comparably high London penetration depth (λ=850 nm [141]) of the material, resulting in lower screening of the external magnetic field inside the superconducting film. In turn, this results in a higher SQUID inductance, *L*. Still, the comparably high normal state resistivity of the material yields a high transfer coefficient dV/dΦ of up to 1300 μV$/\Phi_{0}$, making it a promising candidate for use as a flux-voltage-transducer.

The use of FEBID/FIBID for the fabrication of nanoSQUIDs is in its infancy and many parameters are yet to be optimized. However, the technique offers a vast flexibility and many measures to improve the procedure can be readily implemented in the same single-step approach. The inductance can be possibly reduced by both a thicker film layer in the SQUID loop and a reduced inner loop area, possibly resulting in higher modulation depth and flux sensitivity. In a DB-SQUID, the thickness of the superconducting ring can hereby be tuned independently from the thickness of the DB, resulting in a higher difference in the achievable cross-sectional area between the constriction and the SQUID loop. Furthermore, the effects of a thermal hysteresis commonly found in SNS- and DB-SQUIDs can be mitigated by a resistive shunt of normal conducting W-C or any other normal conducting metal for which a precursor is available. If the shunt is placed on top of the junction, it may also improve the transport of the dissipated heat, further suppressing the thermal hysteresis. Albeit its high λ, the single-step fabrication method makes FIBID/FEBID-grown W-C a promising candidate for the fabrication of nanoSQUIDs due to its high flexibility and resolution.

### 3.5. Other Applications

The previously mentioned applications have been deemed noteworthy enough to merit their own section in this review, yet they are far from being the only ones where FIBID and FEBID finds application for nanopatterning of superconducting devices. Within the restrictions imposed by the precursor of choice and its associated superconducting properties, the technique still finds many different applications. Some last examples are reviewed in this section.

Basset et al. [159] report the usage of He${}^{+}$ FIBID in the fabrication of hybrid Nb/W-C superconducting microwave resonators, exploiting the advantages of He${}^{+}$ FIBID over Ga${}^{+}$ FIBID and FEBID (reduced substrate damage and ion implantation, and improved superconducting properties, respectively). The devices consist of a sputtered Nb thin film, pre-patterned by means of optical lithography, on top of which a superconducting W-C nanowire is placed. Exploring two different approaches (lumped resonator and coplanar waveguide), the authors report appropriate quality factors, large values of kinetic inductance, and high immunity to external applied magnetic fields, which indicates their suitability for the study of spin properties in mesoscopic systems.

Martínez-Pérez et al. [160] report the usage of FIBID for repair and modification of *superconducting* circuits, combining the know-how of circuit edit developed for FIBID with the superconducting properties of W-C grown using a Ga${}^{+}$ FIB. The combined approach of FIB milling and FIBID allowed for the functional modification of a commercial SQUID, replacing the original wiring with newly-grown superconducting W-C deposits. A superconducting Nb device, damaged and rendered inoperable by the effect of an electrostatic discharge, was successfully repaired, replacing the destroyed superconducting and insulating materials with FIBID-grown W-C and SiO${}_{2}$ (from the tetraethylorthosilicate, TEOS, precursor material), respectively. In-situ monitoring of the electrical response of the materials is recommended by the authors, to assess the quality of the modifications and potentially detect any short-circuits appearing from the deposition halo, a thin layer of deposited W-C whose growth is induced by scattered secondary particles and backscattered ions away from the irradiation point.

García-Serrano et al. [161] show how the competing deposition and sputtering effects of FIBID can be exploited to grow a thickness-modulated thin film, whose corrugation defines a potential landscape that pins vortices. Such a periodic modulation in thickness is achieved by sequentially scanning the area in a series of evenly-spaced lines: the substrate undergoes a slight, yet noticeable milling effect along the line through which it is directly irradiated, with the trench being filled with a thicker amount of material, whereas a thinner film gets deposited between lines by the effect of the secondary electrons. The two effects define a continuous film with regular variation of thickness. Owing to the high sensibility that vortices have to minute changes in the film thickness, when the periodicity of the corrugation matches the vortex density imposed by an external magnetic field (a *mode*) [162], vortices tend to remain in their equilibrium positions even under the effect of an externally applied current. Such an effect is experimentally detected through local minima in the magneto-resistance of the film, that would otherwise be higher as unpinned vortices drift along the material. This application exemplifies how side effects of the growth procedure (even those that are potentially unwanted) may find a practical use if the deposition conditions are adjusted accordingly.

As discussed in Section 3.2.3, Dobrovolskiy et al. [125] demonstrated that NbC microstrips fabricated by Ga${}^{+}$ FIBID host ultra-fast vortex motion, which points towards their potential applicability as single-photon detectors. Conventionally, these devices have been designed as narrow (50 nm to 100 nm), meandering nanostripes, with the reduced width being a requirement to ensure the single-photon nature of the sensor and to maintain an adequate sensing efficiency [15]. However, more recent studies indicate that the current density plays a more fundamental role in determining such detection factors [163], therefore loosening the small-width condition that was classically required. As such, the wider microstripes nanopatterned by FIBID do satisfy the requirements for fast detection of individual photons, with the material therefore representing a strong candidate for its fabrication. The authors also remark the possibility of combining the ion-induced growth with the deposition of insulating materials via FEBID to design other advanced functional devices using this material, including superconductor/quantum layered lattices and photonic crystals.

## 4. Conclusions and Outlook

In this review, the fundamentals of focused ion/electron beam induced deposition and their application to the nanopatterning of superconducting materials have been presented. Both techniques, whose applicability for this purpose is restricted to the availability of a suitable precursor, have been reported to yield several superconducting materials containing different elements.

In the case of FIBID, W-C deposited with a Ga${}^{+}$ FIB represents the main source of references in the literature, with recent reports being published on its in-plane and 3D growth using He${}^{+}$ ions instead. Other superconducting materials grown using FIBID are niobium, molybdenum, and carbon. On the other hand, reports on FEBID of superconducting materials include tungsten, molybdenum, and lead.

**Figure 9:**
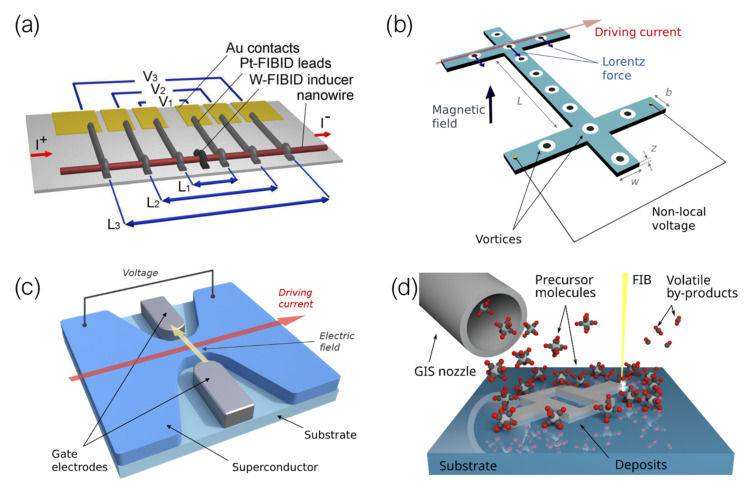
Summary of selected reported phenomena observed in Ga${}^{+}$ FIBID-grown W-C nanostructures outlined in this review: (**a**) Superconducting proximity effect (reprinted from [99], with permissions from AIP Publishing) (**b**) Non-local vortex transport, (**c**) Electric field-induced critical current modulation, and (**d**) SQUID nanofabrication (reprinted from [158]).

Several specific applications have been discussed here, including the superconductivity proximity effect induced by W-C deposits in different materials, the different approaches for the investigation of vortex dynamics in W-C and Nb, including STS and electrical characterization-based approaches, the possibility of tuning the critical current using an external gating voltage, and the utilization of the technique for the fabrication of functional nanoSQUIDs for magnetic sensing (Figure 9).

Overall, this review will have hopefully illustrated the wide range of applicability and potential that the single-step deposition procedures based on irradiation with an FIB and an FEB offer for the research community. With ongoing efforts towards different ion sources and novel precursors, the way is paved towards new and exciting developments in the near future.

## Figures and Tables

**Table 1:** Reported features and characteristic parameters of superconducting materials prepared by FIBID and FEBID. The values of reported critical field are indicated along with the temperature at which they were estimated. In the case of lead, parameters corresponding to the two different superconducting phases detected by the authors are separated by a slash. (*: see text for clarification).

		Met. Cont.	$\rho_{N}$ (μΩ·cm)	$T_{c}$ (K)	$B_{c2}$ (T)	ξ (nm)	λ (nm)	Ref.
Tungsten	Ga${}^{+}$ FIBID	40% to 50%	200 to 300	4.3 to 5.5	9.5 (0 K)	6.25	850	[59,60,61,62,63,64,65]
	2D He${}^{+}$ FIBID	20% to 30%	110 to 240	2.5 to 4.0	4.0 (0.5 K)	6.40	616 to 812	[75]
	3D He${}^{+}$ FIBID	72%	400 to 460	6.4	7.0 to 9.0 (4 K)	4.86 to 5.05	830 to 902	[27]
	FEBID	50%	270	2.0	3.7 (0 K)	—	—	[78]
		26% to 38%	270	4.7 to 5.7	4.0 (1.5 K)	—	830	[79]
Niobium	2D Ga${}^{+}$ FIBID	29%	520	5.4	1 (2 K)	5.94	1101	[13]
	3D Ga${}^{+}$ FIBID	—	380	8.1	8 (4 K)	5.85	471	
Molybd.	Ga${}^{+}$ FIBID	38% to 45%	300 to 600	2.7 to 3.8	2.5 to 4.5 (0 K)	7.6 to 11.2	—	[82]
	FEBID	70%	—	7.2	—	8.00	—	[84]
Carbon	Ga${}^{+}$ FIBID	n/a *	—	7.0	8.8 (0 K)	6.00	—	[85]
Lead	FEBID	40% to 46%	170 to 640	6.6/7.2 *	9.9/0.2 * (0 K)	5.75/40.23 *	—	[86]
